# Recurrent intestinal ischemia following surgery for gastric and duodenal perforations: a case report

**DOI:** 10.1186/s40792-019-0683-9

**Published:** 2019-08-01

**Authors:** Takahiro Korai, Katsunori Kouchi, Ayako Takenouchi, Aki Matsuoka, Kiyoaki Yabe, Chikako Nakata

**Affiliations:** Department of Pediatric Surgery, Tokyo Women’s University Yachiyo Medical Center, 477-96 Ohwada-shinden, Yachiyo City, Chiba 276-8524 Japan

**Keywords:** Extremely low birth weight infant, Intestinal ischemia, Barotrauma

## Abstract

**Background:**

Extremely low birth weight (ELBW) is a risk factor for various gastrointestinal complications. In the recent decades, advances in medicine have increased the survival of ELBW infants with necrotizing enterocolitis (NEC). To our knowledge, there have been no reports of neonates or infants developing simultaneous gastric and duodenal perforations and later developing NEC. We report a case of an extremely low birth weight infant (ELBWI) who developed both gastric and duodenal perforations at the same time and developed NEC after operation for gastric and duodenal perforations.

**Case presentation:**

The patient was a female infant with ELBW who developed both gastric and duodenal perforations at the same time and developed NEC after operation for gastric and duodenal perforations. After birth, endotracheal intubation was performed. However, barotrauma occurred during positive pressure ventilation, resulting in a large area of emphysema in the left lower lung field, leading to collapse of the left lung. This giant bulla may have compressed the pulmonary vein, possibly resulting in pulmonary venous thrombosis (PVT). This episode could have triggered simultaneous gastric and duodenal perforations. In addition, we hypothesized that PVT caused acute arterial ischemia, leading to the development of NEC. The infant was started on heparin for anticoagulation. Later, the infant developed non-immunoglobulin E (IgE)-mediated gastrointestinal food allergies (non-IgE-GI-FAs). The giant bulla associated with barotrauma might have caused PVT, resulting in arterial emboli and multiple simultaneous gastrointestinal perforations.

**Conclusions:**

Anticoagulation therapy with heparin for acute arterial thrombosis is effective for preventing the development of short bowel syndrome. Duodenal and intestinal surgery probably acted as risk factors for the subsequent development of non-IgE-GI-FAs. The infant had been stabilized at the time of writing this report.

## Background

Extremely low birth weight (ELBW) is a risk factor for various gastrointestinal complications. In the recent decades, advances in medicine have increased the survival of extremely low birth weight infants (ELBWIs), including those who develop gastrointestinal complications (e.g., gastrointestinal perforation, necrotizing enterocolitis [NEC], meconium peritonitis) [1]. In neonates, gastric or duodenal perforation is rare [2, 3], and until now, to our knowledge, there have been no reports of neonates or infants developing simultaneous gastric and duodenal perforations and later developing NEC. Here, we present the first reported case of an ELBWI with multiple gastrointestinal ischemic episodes, including simultaneous gastric and duodenal perforations.

## Case presentation

**Fig. 1:**
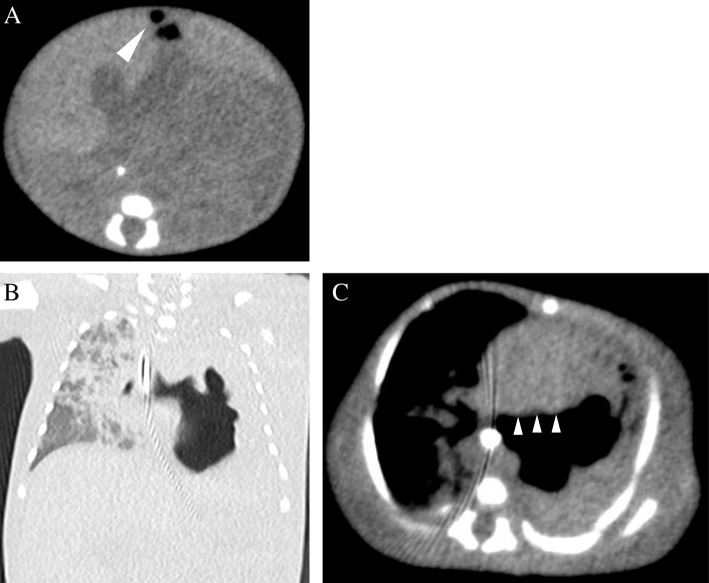
Computed tomography. **a** Mediastinal-conditional computed tomography image showing a small amount of free air (arrowhead). **b** Lung-conditional computed tomography image showing a giant bulla in the left lower lung field. **c** Mediastinal-conditional computed tomography image showing that this bulla was compressing the extrinsic pulmonary vein and left atrium from the dorsal side

**Fig. 2:**
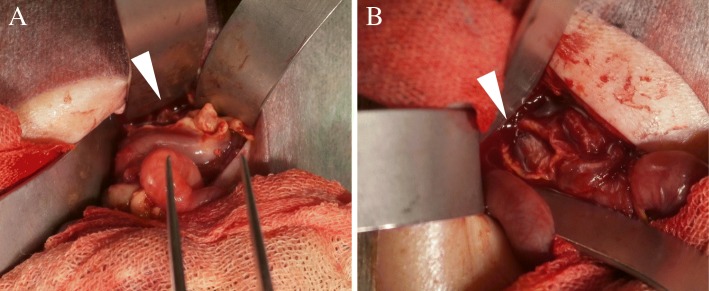
Operation for gastric and duodenal perforations. **a** Gastric perforation of 3 cm at the angulus of the lesser curvature (arrowhead). **b** Duodenal perforation of 1.5 cm at the anterior wall of the duodenal bulb (arrowhead)

**Fig. 3:**
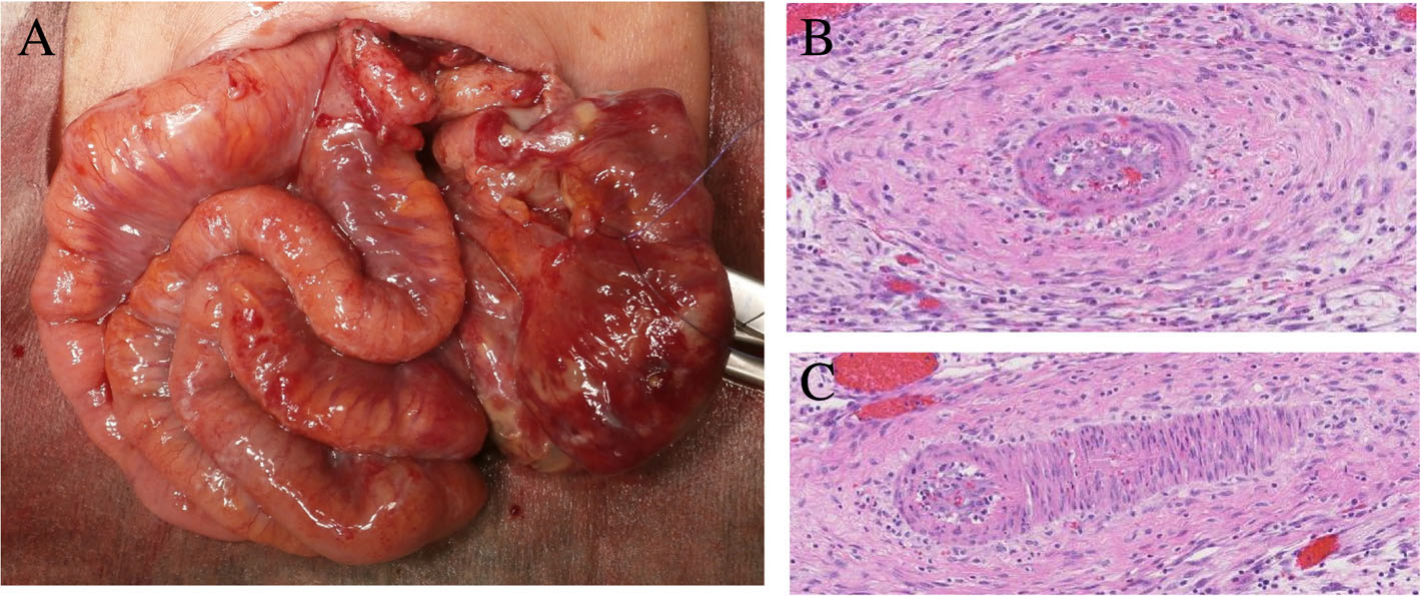
Operation for necrotizing enterocolitis. **a** The color of the mesenteric artery was white, and the whole small intestine was pale. The length of the necrotic jejunum was only 15 cm. **b**, **c** Pathological examination revealed multiple thromboemboli in the mesenteric artery

A female infant was born at 26 weeks of gestation via cesarean delivery because of fetal growth restriction, with a birth weight of 678 g and Apgar scores of 4 and 6 at 1 and 5 min, respectively. She was administered formula on day 0 after birth. On day 2, she passed black feces once. However, as there was no significant change in the general condition, and anemia was absent, no intervention was performed, and follow-up was continued. On day 7, bright red hemorrhage occurred via the oral gastric (OG) tube; radiography showed the tip of the OG tube in the stomach body. On the same day, endotracheal intubation was performed because of frequent apnea, desaturation, and respiratory acidosis on blood gas analysis. The next day, a giant bulla appeared in the left lower lung on chest radiography. On day 9, she suddenly had massive abdominal distention and metabolic acidosis. Abdominal computed tomography showed a tiny amount of free air, and barotrauma occurred due to positive pressure ventilation, resulting in damage to the left bronchus, leading to the development of a large area of emphysema in the left lower lung field and collapse of the left lung (Fig. 1). Gastrointestinal perforation was suspected because of her abdominal findings and free air. The infant underwent laparotomy on the same day. Exploration via a supra-umbilical transverse incision showed a 3-cm gastric perforation at the angulus of the lesser curvature and a 1.5-cm duodenal perforation at the anterior wall of the duodenal bulb. The OG tube was not found in the perforation (Fig. 2). Primary repair of the perforations was performed by suturing in one layer using 6-0 absorbable sutures. On day 22 (postoperative day 13), the infant began an elemental diet (Elental P®; EA Pharma, Tokyo, Japan); the amount of formula was increased by 10 ml each day until the target volume of 120 ml/day was reached. On day 51 (postoperative day 42), she had sudden massive abdominal distention. Fresh blood was aspirated from the nasogastric tube, and abdominal plain radiography showed marked intestinal expansion, pneumatosis intestinalis, and gas in the hepatic portal vein, suggestive of NEC. There was repeated gastrointestinal ischemia, involving the stomach, duodenum, and small intestine. As the large area of emphysema in the left lower lung field and left lung atelectasis were still present, the cause of gastrointestinal ischemia was thought to be PVT. At this point, extensive intestinal ischemia was predicted; therefore, she was started on heparin for anticoagulation. We decided to defer the operation to a later date. Three days later, on day 54, surgery was performed (Fig. 3a). Laparotomy revealed that the mesenteric artery had become white in color. The whole small intestine was pale; however, the length of the necrotic jejunum was only 15 cm. Resection of the necrotic jejunum was performed, followed by jejunostomy. The length of the remaining small intestine was approximately 60 cm. Pathological examination revealed multiple thromboemboli in the mesenteric artery (Fig. 3b).

**Fig. 4:**
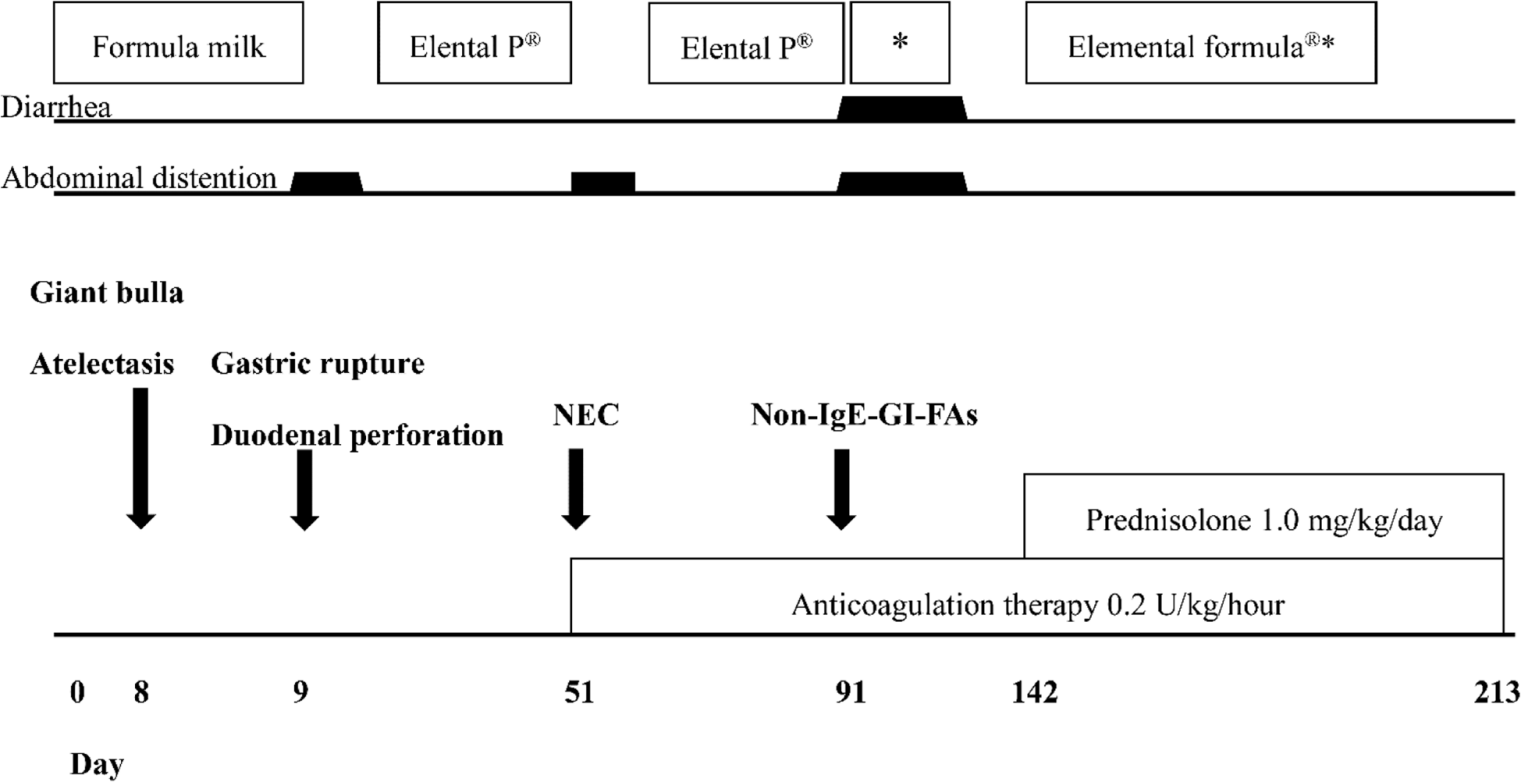
Clinical course and management of recurrent intestinal ischemia. Clinical course and management of recurrent intestinal ischemia in an extremely low birth weight infant

As the presence of thrombus had been proven pathologically, we decided to continue anticoagulation therapy. On day 64, the infant was administered an elemental diet and underwent infusion of intestinal fluid collected from the proximal jejunostomy stoma into the distal jejunostomy to prevent nutritional and hepatic disorders and atrophy of the intestinal villi. On day 73, closure of the jejunostomy was performed. On day 91, there was sudden abdominal distention and diarrhea. After this episode, the C-reactive protein (CRP) level increased to 4.29 mg/dl. T cell-mediated non-IgE-GI-FAs, which occur in newborns and infants who are introduced to milk protein, were suspected because of abdominal distention after the introduction of the milk, elevated CRP levels, and her history of duodenal and small intestinal surgery. For diagnostic and therapeutic purposes, an elemental diet (Elemental Formula®; Meiji, Tokyo, Japan) was administered. The abdominal symptoms were prolonged; therefore, we tried improving these symptoms by keeping the infant non per os. On day 142, after improvement of the abdominal symptoms, administration of Elemental Formula® was resumed in combination with prednisolone (1.0 mg/kg/day). She was diagnosed as having non-IgE-GI-FAs because of the lack of recurrence of abdominal symptoms (Fig. 4). Currently, the infant’s condition has stabilized without prednisolone, and she has been receiving Elemental Formula®.

## Discussion

Gastric and duodenal perforations are rare in neonates [2, 3]. The risk factors for duodenal perforation include low birth weight (LBW) with asphyxia, ischemic stress, and presence of a nasojejunal feeding tube [3], whereas the risk factors for neonatal gastric perforation include ischemic stress and iatrogenic causes, such as nasogastric tube insertion [2]. To our knowledge, there are no reports of gastric and duodenal perforations occurring simultaneously as observed in the patient described in this report. In our case, the gastric perforation was considered to be caused by ischemic stress and not iatrogenic stress because the tip of the OG tube was found to be in the stomach body on the radiograph, and the OG tube was not observed in the perforation during surgery. We hypothesize that the first ischemic episode, which resulted in simultaneous gastric and duodenal perforations, was caused by hypoxia and PVT.

PVT is rare, and its causes are reported to be trauma [4], lobectomy, and lung transplantation [5]. There are some reports of PVT being associated with large hiatal hernias [6] and bronchogenic cysts [7], both of which are intrathoracic lesions. Giant intrathoracic lesions and giant bullae in the left lobe may cause dorsal compression of the extrinsic pulmonary vein and left atrium, which may then lead to PVT. There are no reports of PVT related to giant bullae associated with barotrauma. The giant bulla observed in our case might have caused PVT. During the second ischemic episode, pathological examination revealed features of NEC, which was considered to have been triggered by PVT, along with multiple thromboemboli in the mesenteric artery. As PVT was considered to be caused by giant intrathoracic lesions compressing the left atrium for a long time, puncture or drain placement should have been performed in the giant bulla. However, a limitation of this report is that contrast-enhanced CT was not performed for the infant; therefore, we could not detect the pulmonary thrombus in the left atrium.

After the development of NEC, we initiated heparin-based anticoagulation therapy because we predicted extensive intestinal ischemia. According to the algorithm for anticoagulation selection for pediatric venous thromboembolism by age, heparin is selected for patients less than 1 year of age [8]. We speculate that the administration of heparin at 0.2 U/kg/h may have resulted in the shrinkage of the thrombus and subsequent improvement in the blood flow in the intestines. In cases involving extensive intestinal ischemia due to arterial thrombus, anticoagulation therapy should be considered to prevent the development of short bowel syndrome.

The diagnostic criteria for non-IgE-GI-FAs were as follows: (1) symptoms, such as abdominal distention, diarrhea, and vomiting following the administration of milk protein; (2) disappearance of these symptoms on the elimination of milk protein; and (3) no recurrence of the symptoms after the introduction of the therapeutic formula without milk protein, such as Elemental Formula®. An elevation in the CRP levels is supportive of a diagnosis of non-IgE-GI-FAs [9, 10]. We reported that full-layer surgical invasion of the duodenum and small intestine is a risk factor for non-IgE-GI-FAs [10]. As a risk factor was detected, the third ischemic episode was thought to be caused by non-IgE-GI-FAs.

## Conclusion

An intrathoracic lesion, such as the giant bulla, could have caused PVT, resulting in arterial emboli and multiple simultaneous gastrointestinal perforations. Gastrointestinal surgery predisposes neonates to developing non-IgE-GI-FAs; therefore, careful follow-up is crucial in such patients.

## Data Availability

The dataset supporting the conclusions of this article is included within the article.

## Abbreviations

ELBW: Extremely low birth weight
ELBWIs: Extremely low birth weight infants
IgE: Immunoglobulin E
LBW: Low birth weight
NEC: Necrotizing enterocolitis
Non-IgE-GI-FAs: Non-immunoglobulin E-mediated gastrointestinal food allergies
OG: Oral gastric
